# Supplementary material for: Integrated analysis of the aqueous humor microbiome and lens capsule transcriptome in high myopia cataract: a pilot study
Source: Front Med (Lausanne). 2026 Jun 16;13:1845205. doi: 10.3389/fmed.2026.1845205 (PMC13314463; doi:10.3389/fmed.2026.1845205)
Supplement: Supplementary file 3 [file Data_Sheet_1.zip › 3.Beta_Diversity/NMDS/C372089/NMDS_bray_curtis_3D.pdf]

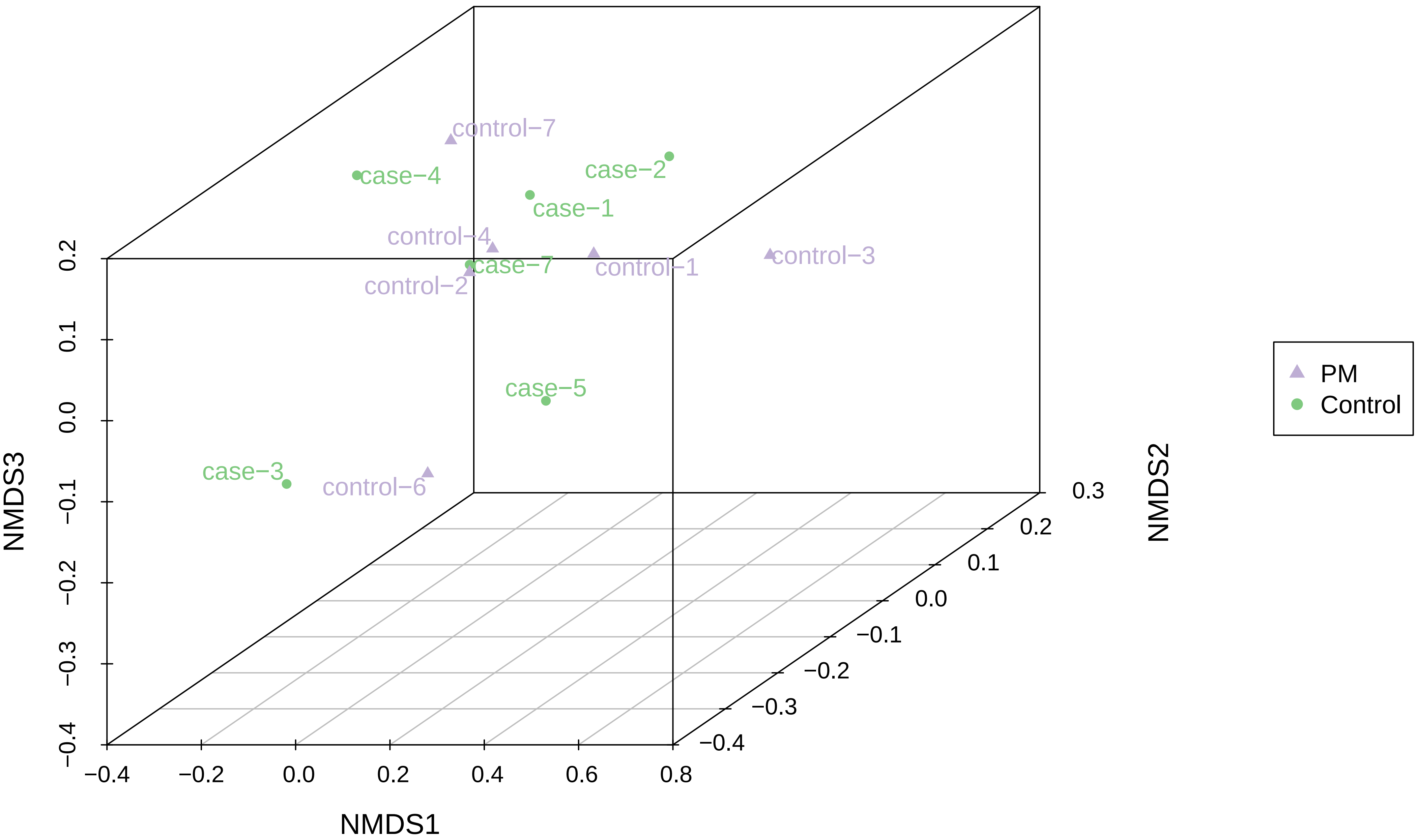
control−7
case−4
case−2
case−1
control−4
case−7
control−1
control−3
control−2
case−5
case−3
control−6
PM
Control
NMDS1
NMDS2
NMDS3
−0.4
−0.2
0.0
0.2
0.4
0.6
0.8
0.3
0.2
0.1
0.0
−0.1
−0.2
−0.3
−0.4
0.2
0.1
0.0
−0.1
−0.2
−0.3
−0.4
